# Variant Biliary Anatomy in Biological Siblings

**DOI:** 10.7759/cureus.34199

**Published:** 2023-01-25

**Authors:** Murali Dharan, Eric Vecchio

**Affiliations:** 1 Department of Gastroenterology and Hepatology, University of Connecticut Health, Farmington, USA

**Keywords:** familial, duct, bile, posterior, right, aberrant

## Abstract

Laparoscopic cholecystectomy is the standard of care for cholecystolithiasis but carries an increased risk of biliary injury compared to open cholecystectomy. Complications from laparoscopic cholecystectomy can be related to several factors. These include - (i) technical factors that depend on the skill of the surgeon, (ii) pathologic factors such as associated inflammation and adhesions, and (iii) anatomic factors such as biliary anatomy. Aberrant biliary anatomy is a major cause of bile duct injury during surgery. To the best of our knowledge familial aberrant biliary anatomy has not been previously reported in the literature. We report a case series of two biological sisters with isolated posterior right duct syndrome and present a brief literature review of this medical condition.

## Introduction

Variant biliary anatomy is a well-recognized risk factor for bile duct injury during laparoscopic cholecystectomy [1,2]. Despite its prevalence in up to 25% of patients, variant biliary anatomy and resultant inadvertent biliary injuries cause considerable morbidity for patients and can delay diagnosis of post-operative bile leak [3]. The most common source of aberrant ducts is from the right hepatic lobe which is usually the sole source of bile drainage for the liver segments they course from [4,5]. Aberrant right posterior bile duct has been reported in up to 6% of the general population in the West. In contrast, with normal biliary anatomy, the tertiary and secondary biliary radicals in the right and left lobes of the liver drain into the right and left hepatic ducts, respectively, which join to form the common hepatic duct that becomes the common bile duct following cystic duct insertion. We present cases of two biological sisters where each of them had aberrant isolated right posterior duct, which may suggest a familial component to variant biliary anatomy.

## Case presentation

Case 1

A 31-year-old female with a family history of gallbladder disease necessitating cholecystectomy (mother and sister) presented with exacerbation of chronic intermittent right upper quadrant abdominal pain. She was found to have elevated liver chemistries (aspartate aminotransferase {AST} 437 U/L, alanine aminotransferase {ALT} 267 U/L, and alkaline phosphatase {ALP} 136 U/L, total bilirubin 2.1 mg/dL), lipase of >6000 U/L, and a prominent 7 mm bile duct (Table 1). Following a diagnosis of gallstone pancreatitis, she underwent laparoscopic cholecystectomy without intra-operative cholangiogram (IOC).** **The patient was readmitted 10 days later for nausea, vomiting, and epigastric abdominal pain. Her liver chemistries were found to be elevated to a similar degree with a total bilirubin of 2.3 mg/dL and lipase >1000 U/L compared to normal labs prior to discharge.

**Table 1:** Lab values on initial presentation, readmission, and after ERCP. ERCP: endoscopic retrograde cholangiopancreatogram

Chemistry (with normal reference range)	At initial presentation	At readmission	After ERCP
AST (aspartate aminotransferase) 17-35 U/L	439	258	71
ALT (alanine aminotransferase) 8-39 U/L	517	356	224
ALP (alkaline phosphatase) 39-113 U/L	136	219	190
Total bilirubin 0.2-1.2 mg/dL	2.3	1.5	0.6
Lipase 8-51 U/L	> 6000	1670	-

**Figure 1:**
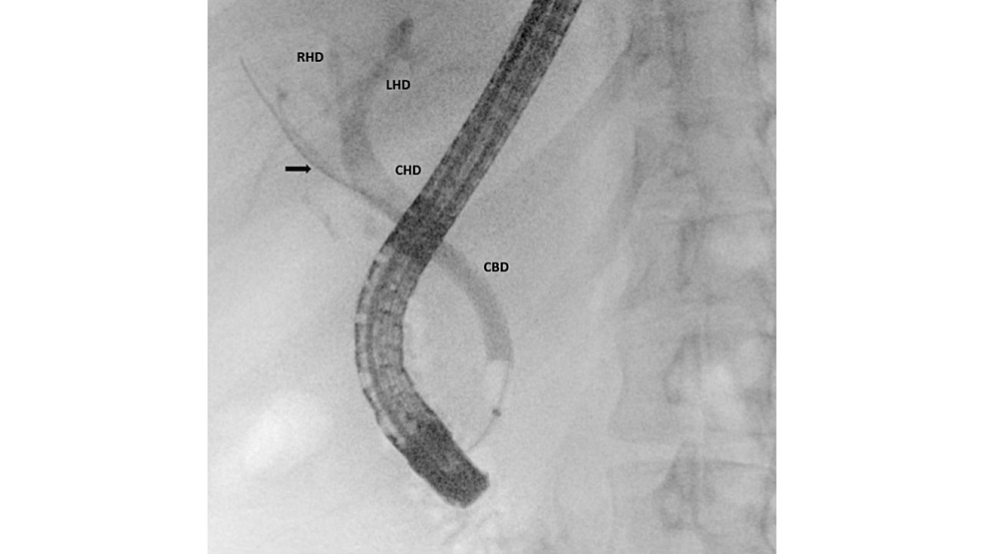
Cholangiogram showing variant biliary anatomy in Case 1 with a guidewire in the right posterior duct which directly joins the common hepatic duct (arrow). RHD: right hepatic duct; LHD: left hepatic duct; CHD: common hepatic duct; CBD: common bile duct

Given the suspicion of choledocholithiasis, the patient underwent endoscopic retrograde cholangiopancreatogram (ERCP)with biliary sphincterotomy followed by biliary stone and sludge extraction. During occlusion cholangiogram, variant biliary anatomy was noted with an isolated right posterior duct draining from segment 6 draining directly into the common hepatic duct, separate from the confluence of the right and left hepatic ducts (Figure 1). She was discharged subsequently and is currently doing well.

Prior to the endoscopic intervention, the patient mentioned history of complicated cholecystectomy in her older sister, who was also taken care of by the author, four years ago. The patient’s sister’s case history is presented below.

Case 2

**Figure 2:**
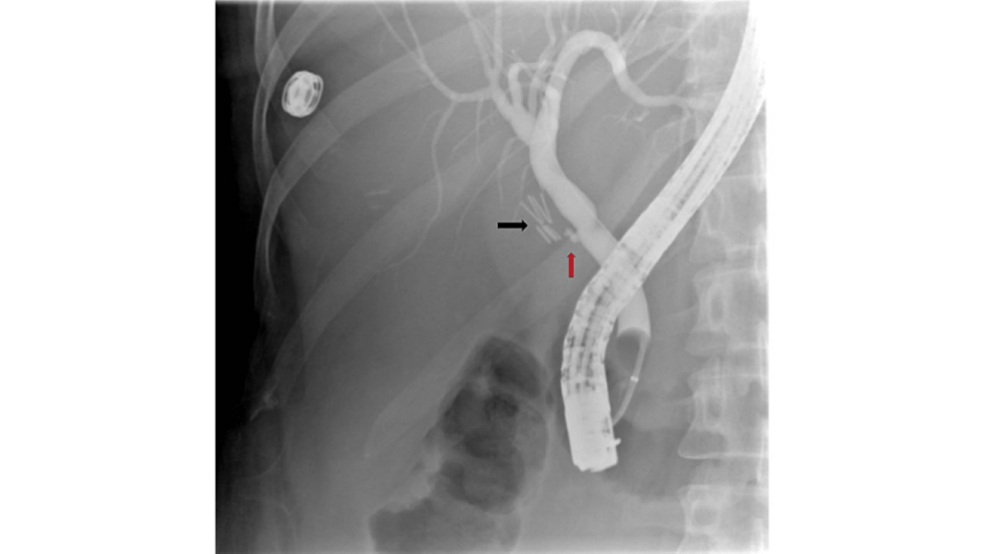
Occlusion cholangiogram without evidence of bile leak in Case 2. Black arrow points to cholecystectomy clips and red arrow points to cystic duct remnant.

A 32-year-old woman on medication for chronic back pain underwent outpatient surgical evaluation for chronic postprandial nausea and epigastric discomfort radiating to the right upper quadrant and interscapular region. The patient underwent an elective laparoscopic cholecystectomy with intra-operative findings of chronic cholecystitis and a normal intra-operative cholangiogram (IOC). The post-operative course was complicated by significant abdominal pain, nausea, and vomiting refractory to conservative management. She was readmitted with abdominal distention and fever of 101°F. A hepatobiliary iminodiacetic acid (HIDA) scan was performed and demonstrated a bile leak. Endoscopic retrograde cholangiopancreatography (ERCP) with biliary sphincterotomy was pursued and a 10 French by 7 cm trans-papillary biliary stent was placed. Cholangiogram demonstrated a non-dilated normal appearing biliary system without contrast extravasation. Due to persistent symptoms, a right upper ultrasound was performed revealing a small 1.7 cm fluid collection in the gallbladder fossa, which was not amenable to percutaneous drainage. She was discharged after optimization of pain management but represented six days later with symptom relapse. Computed tomography (CT) scan with intravenous contrast was performed, which demonstrated two communicating collections along the inferior margin of the left lobe of the liver consistent with biloma. Induration and more complex dense collections were found along the right lobe of the liver along segments 5 and 6 thought to represent blood and bile. Percutaneous drainage was performed but repeat HIDA, unfortunately, demonstrated continued bile leak. Her percutaneous drain was removed four days later when CT demonstrated a reduction in size of collection. She continued to have persistent right upper quadrant (RUQ) pain. Follow-up ERCP was performed to remove the common bile duct (CBD) stent. The cholangiogram was normal without bile leak (intra-procedural radiology consultation concurred with findings) and some sludge was swept from the duct (Figure 2).

**Figure 3:**
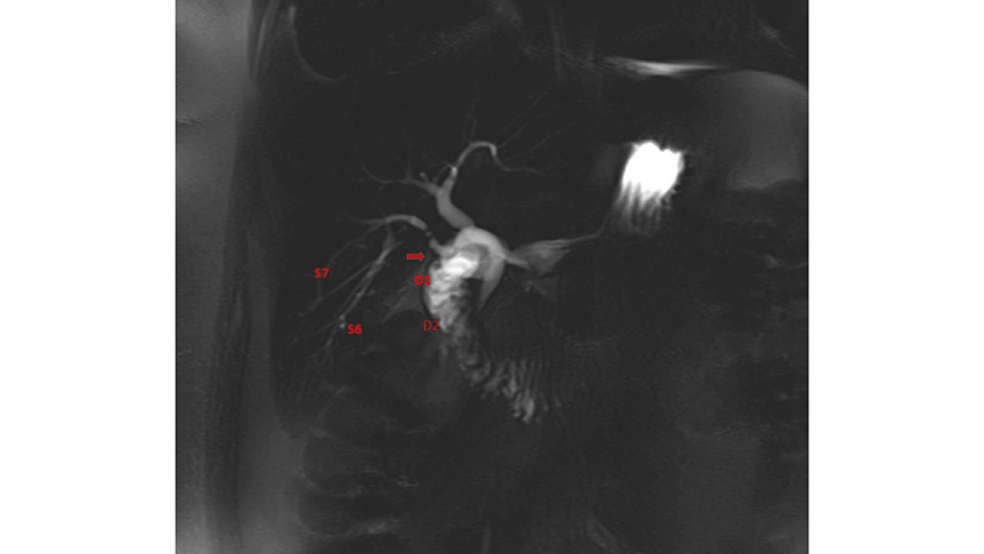
Biliary fistula between hepatic segment 6/7 and duodenum in Case 2 (arrow). S6: hepatic segment 6; S7: hepatic segment 7; D1: first portion of duodenum; D2: second portion of duodenum

A month later the patient underwent magnetic resonance imaging (MRI) study with contrast for persistent symptoms, which demonstrated a fistulous connection between hepatic segments 6 and 7 and the duodenal cap. This was likely a consequence of inadvertent bile duct injury during surgery, related to variant biliary anatomy, specifically an isolated right posterior duct (Figure 3). The patient chose to transfer care to another hospital and underwent segmental hepatic resection and fistula closure with symptom resolution.

## Discussion

Bile duct injury is a serious complication of laparoscopic cholecystectomy and occurs with misidentification of the anatomy by the surgeon or due to variations in biliary anatomy [1,2]. Aberrant right hepatic ducts are the most common anatomic variants of the biliary tree [3].

Normally the right posterior bile duct joins the right anterior bile duct to form the right hepatic duct. Anomalous drainage of the right posterior bile duct has been reported in 2-5% of patients undergoing peri-operative cholangiograms [6]. It is important to note the possible insertion points of an aberrant right posterior hepatic duct mainly include the gallbladder, the cystic duct, and the common hepatic duct [3].

The diagnosis of bile duct injury can be challenging, especially in the setting of variant anatomy. There can be delay in recognition of the injury and initiation of appropriate management, as it did in our patient (Case 2). Traditional ERCP with stenting to establish low-pressure drainage into the duodenum may not be successful since the aberrant bile duct may not communicate with the biliary system. In a small retrospective study comparing patients with aberrant biliary anatomy to patients with normal anatomy, a statistically significant delay in mean days to diagnosis of bile leak was noted (29 days, p<0.005) [2].

If an aberrant duct leak is suspected and not found on ERCP or magnetic resonance cholangiopancreatogram (MRCP), retrograde cholangiogram via injection of a mature biloma showed success in localizing the leak in the same small case series [2]. If a leak is detected, management varies depending on the patient’s symptoms and location of the leak. Conservative non-operative management can be successful in up to 50% of the cases when the diagnosis is made early [7]. If the leak persists eight weeks after bile duct injury, Roux-en-Y hepaticojejunostomy (often complicated by anastomotic strictures), partial hepatectomy of the affected lobe, or interventional radiology approaches including hepatic atrophy induction have been described [3,7-9]. While it can be challenging in a non-dilated biliary system, the placement of a percutaneous trans-hepatic catheter into the injured bile duct prior to surgery could help surgical repair - presumably by aiding intra-operative delineation of biliary anatomy [10]. When variant biliary anatomy is recognized pre-operatively, placement of endoscopic naso-biliary drainage can sometimes reduce intra-operative and post-operative complications [1].

Although aberrant right posterior duct anatomy is the most common variant of biliary anatomy and has been noted in up to 5% of the general population in the West, routine pre-operative biliary imaging is currently not the standard of care [6,11-13].

Embryological development of intra-hepatic biliary system begins with phenotypic change of hepatoblasts towards cholangiotypic cells followed by remodeling of the ductal plate structure and further maturation of tubular ducts [14]. Remodeling of the intrahepatic bile ducts is not complete at birth and continues to occur in the early post-natal phase [14]. There is close interaction between the developing intra-hepatic biliary system and vascular systems of the portal and hepatic arterial circulation [14]. It is conceivable that genetic factors may play a significant role in the embryological development of the intra-hepatic biliary system.

Though the differences in post-operative course are striking, the presence of aberrant biliary anatomy in two blood-related sisters is intriguing - Case 1 was noted to have isolated right posterior duct inserted into the common hepatic duct on cholangiogram, and while the exact insertion of the right posterior duct in Case 2 cannot be ascertained, she likely sustained injury to this duct during cholecystectomy. While this raises the possibility of familial component to variant biliary anatomy, more studies are required. Although case reports and case series describing variant biliary anatomy are common and date back to the early 1990s, to the best of our knowledge, this is the first study (of two cases) describing blood relatives having similar variant biliary anatomy. It highlights the importance of taking a careful family history in patients with biliary disease and cognizance of variant biliary anatomy. Perhaps the variant biliary anatomy was recognized in the younger sibling prior to cholecystectomy resulting in a better operative outcome.

## Conclusions

Given the low overall incidence of aberrant right posterior bile duct anatomy routine pre-operative imaging to delineate biliary anatomy is generally not performed prior to cholecystectomy. Though infrequent, presence of variant biliary anatomy can increase risk of bile duct injury at cholecystectomy. A family history of complicated biliary surgery should prompt careful evaluation of biliary anatomy prior to initiating endoscopic or surgical biliary therapy. Our study raises the intriguing question - can aberrant biliary anatomy sometimes be familial?

Embryological development of intra-hepatic biliary system outlined in this study suggests the possibility of familial predisposition to biliary anatomy and may explain the similar nature of aberrant biliary anatomy in the two biological sisters we have reported on. Further studies involving larger and more robust databases can help shed light on the possibility of familial predisposition to aberrant biliary anatomy.
